# Structured, person-centred glycaemic optimisation before surgery: The Manchester IP3D experience

**DOI:** 10.1016/j.clinme.2026.100619

**Published:** 2026-07-23

**Authors:** Mohammed S.O. Ahmed, Mariyah Ahmed, Adhithya Sankar, Jacqueline Toombs, Hood Thabit

**Affiliations:** a Diabetes, Endocrine and Metabolism Centre, Manchester Royal Infirmary, Manchester University Hospitals NHS Foundation Trust/University of Manchester, Manchester, UK; b Division of Diabetes, Endocrinology and Gastroenterology, School of Medical Sciences, Faculty of Biology, Medicine and Health, University of Manchester, Manchester, UK; c Department of Diabetes, Endocrinology and Obesity Medicine, Salford Royal Hospital, Northern Care Alliance NHS Foundation Trust, Salford, UK

**Keywords:** Perioperative diabetes care, Diabetes optimisation, Diabetes specialist nurse, Elective surgery

## Abstract

**Study objective:**

To evaluate the impact of a diabetes specialist nurse (DSN)-led perioperative optimisation service on glycaemic control in adults undergoing elective surgery.

**Design:**

Retrospective service evaluation.

**Setting:**

Manchester Royal Infirmary (Manchester University NHS Foundation Trust), a large UK teaching hospital and regional specialist centre.

**Participants:**

398 adults with diabetes (mean age 62.2 years; 76.3% type 2 diabetes) referred to the preoperative pathway between 2020 and 2024.

**Interventions:**

A structured pathway involving DSN-led outpatient consultations, blood result monitoring, and targeted education to support self-management and guideline adherence.

**Main outcome measure(s):**

Change in HbA1c from referral to the perioperative period (within 12 weeks of surgery) and predictors of glycaemic improvement.

**Results:**

Participants had a median baseline HbA1c of 75 mmol/mol. Over a median 16-week interval with two DSN contacts, HbA1c decreased by a median of 18 mmol/mol. Multivariable analysis identified higher baseline HbA1c (p < 0.001) and frequency of DSN contacts (p < 0.001) as independent predictors of improvement. Notably, medication intensification and diabetes technology use were not significantly associated with HbA1c reduction.

**Conclusions:**

A structured DSN-led pathway achieves clinically meaningful HbA1c reductions in high-risk surgical patients. These gains were associated with specialist engagement rather than complex medication adjustments, suggesting that focused education is a safe, effective strategy for perioperative optimisation that minimises pharmacological burden in the secondary care setting.

## Highlights

• **Effective risk mitigation:** A nurse-led pathway cut median HbA1c by 18 mmol/mol, lowering a key surgery risk.
• **Behavioural driver:** Improvement occurred without drug or technology changes, proving specialist support drives success.
• **Social equity:** Strong outcomes in deprived areas prove the service successfully reaches underserved groups.
• **Predictors of success:** Frequent specialist nurse contact was the top predictor of improvement, highlighting MDT value.
• **Scalable service model:** This study offers a practical framework for secondary care to meet national standards.

## Introduction

Diabetes is a common comorbidity in surgical populations, affecting up to one in six individuals undergoing elective procedures in the UK.^1^ It is associated with increased perioperative complications, including infection, delayed wound healing, cardiovascular events and prolonged hospital stay.^2^, ^3^, ^4^, ^5^ Poor glycaemic control remains a key modifiable risk factor, yet many patients present for surgery with HbA1c levels exceeding recommended thresholds.^1^

National initiatives, including the NHS Getting It Right First Time (GIRFT) programme and Centre for Perioperative Care (CPOC) guidance, emphasise structured perioperative pathways and safe preoperative glycaemic targets.^1^, ^6^ While DSN-led interventions improve glycaemic outcomes in other settings, their role in preoperative optimisation remains under-evaluated.^1^

To address this gap, the Improving the Perioperative Pathway of Patients with Diabetes (IP3D) project was established at Manchester Royal Infirmary in 2020.^7^ This evaluation examined the impact of a DSN-led preoperative optimisation pathway on glycaemic control in people with diabetes referred prior to elective surgery. We evaluated changes in HbA1c, diabetes technology use and predictors of glycaemic improvement to inform perioperative diabetes care models within the NHS.

## Methods and materials

This retrospective service evaluation project examined the DSN-led preoperative pathway at Manchester Royal Infirmary, a major trauma centre with 770-bed capacity. Adults with diabetes referred for diabetes optimisation prior to elective surgery between August 2020 and August 2024 were eligible for inclusion. A total of 704 patients were identified via a DSN-maintained database; 398 were included after excluding those without diabetes, without DSN contact, or who underwent surgery before DSN review. This project was registered with the hospital clinical audit department. As a service evaluation project, formal ethical approval was not required.

### Intervention

A perioperative DSN-led service was established, accepting referrals and providing care for people with diabetes undergoing elective surgery through outpatient consultations and perioperative inpatient review. Each patient received an initial DSN-led outpatient consultation comprising: (1) structured diabetes education addressing perioperative self-management, sick-day rules and medication management around the time of surgery; (2) review and optimisation of diabetes medications where clinically indicated; (3) assessment of diabetes technology use and initiation of continuous glucose monitors (CGMs) where appropriate; and (4) a personalised care plan with defined glycaemic targets. Follow-up DSN contacts were arranged based on clinical need. The DSN also undertook morning reviews of the elective admissions list, daily ward rounds for urgent inpatient interventions and afternoon outpatient clinics, along with targeted education for clinical staff to support adherence to perioperative diabetes pathways.

### Data collection

Data extracted from the hospital’s electronic patient records included demographics, index of multiple deprivation (IMD), type of diabetes, diabetes medications, use of diabetes technology (CGMs, insulin pumps, hybrid closed loop systems (HCL)), number of DSN contacts and HbA1c measurements.

Baseline HbA1c was defined as the value recorded at referral to the optimisation pathway. Perioperative HbA1c is the value obtained within 12 weeks prior to surgery or, when not available, the most recent HbA1c within 12 weeks following surgery. Socioeconomic status was determined using the IMD decile from the English Indices of Deprivation 2019, where 1 indicates the most deprived 10% of areas.^8^

### Outcomes

The primary outcome was the change in HbA1c between baseline and the perioperative period. Secondary outcomes included changes in diabetes medications, trends in diabetes technology use, and associations between clinical or demographic factors and HbA1c improvement.

### Statistical analysis

Data were analysed using SPSS v29.0 (IBM, USA) applying complete case analysis. Normality was assessed using the Kolmogorov–Smirnov test. Continuous variables were summarised as mean ± SD for normally distributed data or as median (interquartile range (IQR)) for non-normally distributed data.

Between-group comparisons were performed using independent sample *t*-tests for parametric data or Mann–Whitney *U* test for non-parametric data.

Correlations were examined using Pearson’s correlation coefficient for parametric data and Spearman’s rank correlation for non-parametric data.

Variables associated with HbA1c change in univariate linear regression (*p* < 0.1) were entered into a multivariate model; predictors with *p* < 0.05 were retained. Model fit was evaluated using adjusted *R*².

## Results

Baseline clinical characteristics are summarised in Table 1. The mean age was 62.2 ± 13.1 years and mean BMI was 29.8 ± 6.9 kg/m². The majority had type 2 diabetes (76.3%), followed by type 1 diabetes (18.4%) and type 3c diabetes (5.3%). Median baseline HbA1c was 75 mmol/mol; IQR 63–89 with only 32.7% achieving the recommended glycaemic target of ≤69 mmol/mol for surgical optimisation at referral. The cohort predominantly originated from the most deprived third of the UK population (IMD decile median 3; IQR 2–6). Use of diabetes technology was limited; 23.4% were on CGM. 5.3% used insulin pumps and 1.3% used HCL. A majority of HbA1c values were preoperative (62.6%, n = 191), while 22% (n = 67) were measured within 12 weeks postoperatively.

**Table 1:** Demographics and clinical characteristics of study population.

Patient characteristics (n = 398)	
Age (years)^a^	62.2 ± 13.06
Weight (kg)^a^	85.6 ± 21.4
BMI (kg/m^2^)^a^	29.8 ± 6.89
Baseline HbA1c (mmol/mol)^b^	75.0 (63.2–88.8)
HbA1c to target % (≤69 mmol/mol)^c^	32.7
Sex^c^	
• Male	63.1
• Female	36.9
Ethnicity (%)^c^	
• White	72.6
• Black	10.8
• Asian	8.0
• Mixed/Other	8.6
Diabetes type (%)^c^	
• Type 1	18.4
• Type 2	76.3
• Type 3c	5.3
IMD decile^b^	3 (2–6)
Insulin pump (%)^c^	5.3
HCL (%)^c^	1.3
CGM (%)^c^	23.4

IMD, Index of Multiple Deprivation; CSII, continuous subcutaneous insulin infusion; HCL, hybrid closed loop; CGM, continuous glucose monitoring.

a Data are mean±standard deviation.

b Data are median (IQR).

c Data are number (%) of patients.

The primary outcome, HbA1c change from baseline to perioperative period, presented a significant median HbA1c reduction of −18 mmol/mol; IQR −43 to −4. The median interval between baseline and repeat HbA1c measurement was 16 weeks (IQR 7.4–30.4) and median number of DSN contacts was 2 (IQR 1–4). Additional main outcomes are summarised in Table 2.

**Table 2:** Clinical characteristics following DSN review.

Characteristics (n = 398)	Values
Change in HbA1c mmol/mol (IQR)^a^	−18.0 (−43.0 to −4.0)^b^
HbA1c to target %; ≤69 mmol/mol	50.5
Repeat HbA1c interval (weeks)^a^	16.0 (7.4–30.4)
Medication change (%)^c^	52 (13.1)
New CGM sensor start (%)^c^	25 (6.3)
Number of DSN visits^a^	2 (1–4)

a Data are median (IQR).

b Negative value indicates improvement.

c Data are number (%) of patients.

Before the preoperative intervention, most patients were managed with oral hypoglycaemic agents, either as monotherapy (35.0%) or in combination with insulin (22.3%). Following intervention, insulin use increased modestly by 0.7%. (Table A2 in the supplementary file). Overall, medication changes did not significantly affect the multivariable model (p = 0.982).

Twenty-five patients (6.3%) were initiated on CGM during the preoperative DSN review. CGM initiation was not significantly associated with HbA1c change (p = 0.698). CGM starters tended to have higher levels of socioeconomic deprivation, as reflected by their IMD decile. However, no significant differences were observed in DSN contact frequency, medication change (Table 2) or outcomes across IMD deciles (Table 1).

### Correlation analysis

Significant correlations were observed between change in HbA1c and several measured variables (Table 3). HBA1c change was expressed as a negative value to denote reduction, preserving the directionality of correlation coefficients. Baseline HbA1c showed a moderate negative correlation with change in HbA1c (ρ = −0.244, *p* < 0.001), indicating greater improvement among patients with higher baseline HbA1c levels. Repeat HbA1c values were positively correlated with HbA1c change (ρ = 0.328, *p* < 0.001). No significant associations were observed between HbA1c change and age, BMI, deprivation score, or number of DSN contacts in univariate analysis (Table 3). The lack of univariate association between DSN contacts and HbA1c change likely reflects confounding by baseline HbA1c levels; however, this was accounted for in the multivariable analysis below.

**Table 3:** Correlation between clinical and demographic variables and change in HbA1c.

Variable	Spearman's ρ	p-value
Baseline HbA1c*	−0.244	<0.001
Repeat HbA1c result*	0.328	<0.001
HbA1c repeat interval (weeks)*	−0.194	<0.001
Age	−0.037	0.463
BMI	0.019	0.715
IMD rank	0.039	0.440
IMD decile	0.045	0.374
Number of nurse visits	−0.017	0.741
Preoperative interval (weeks)	0.003	0.963

* Statistically significant.

### Predictors of HbA1c improvement

**Fig. 1:**
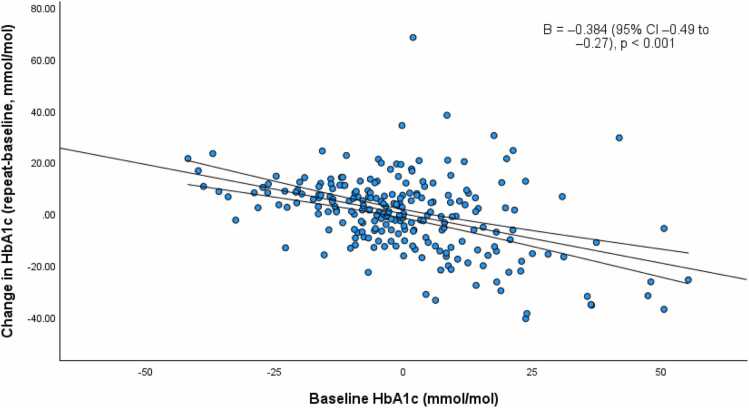
Association between baseline HbA1c and change in HbA1c (negative value indicates improvement).

**Fig. 2:**
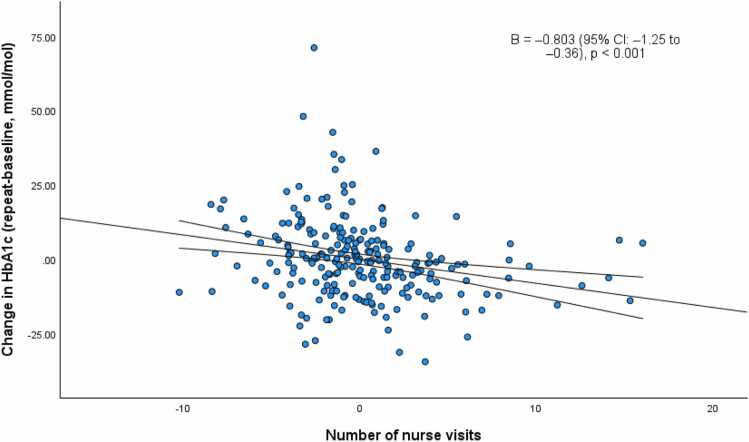
Association between number of nurse visits and change in HbA1c (negative value indicates improvement).

In multivariable analysis, higher baseline HbA1c (B = −0.384, p < 0.001) and greater DSN contact frequency (B = −0.803, p < 0.001) independently predicted HbA1c reduction (Fig. 1 and 2). Other demographic, socioeconomic, medication, and technology variables were not significant predictors (Table A2 in the supplementary file). The final multivariable model explained 33.9% of the variance in HbA1c change (adjusted R² = 0.339).

## Discussion

This service evaluation demonstrates that a structured, DSN-led perioperative optimisation pathway can achieve a clinically significant HbA1c reduction (median 18 mmol/mol) within a 16-week window. The primary significance lies in the modifiability of surgical risk; by transforming ‘high-risk’ patients into viable surgical candidates, such pathways directly address elective backlogs and perioperative safety. Importantly, these improvements occurred without significant changes to pharmacotherapy or increased technology use, highlighting the impact of DSN-led engagement. Our findings align with national recommendations from CPOC and the GIRFT programme, both of which advocate proactive glycaemic optimisation before surgery.^1^, ^6^ The Royal College of Physicians (RCP), GIRFT and CPOC have placed increasing emphasis on ‘prehabilitation’ and integrated care; our results provide a practical, evidence-based framework for these mandates.

Two independent factors for HbA1c reduction were identified: higher baseline HbA1c and greater frequency of DSN contacts. Baseline HbA1c was the strongest determinant of improvement, consistent with previous evidence that individuals furthest from target benefit most from structured support.^3^, ^9^ The association between HbA1c improvement and DSN contact highlights the importance of early referral, continuity of engagement, and adequate lead-in time before surgery. Furthermore, socioeconomic deprivation and technology use were not predictive of glycaemic improvement. In contrast to wider diabetes literature, this suggests that structured DSN input may mitigate socioeconomic disparities in perioperative care.^10^, ^11^ The absence of a significant impact from CGM use likely reflects short adaptation periods and selective initiation among complex cases, emphasising that technology alone is insufficient without accompanying behavioural support. However, only 25 patients (6.3%) commenced CGM and this study was not powered to detect a technology-specific effect.

Medication changes were infrequent and not significantly associated with HbA1c change, possibly reflecting a pragmatic approach to avoid hypoglycaemia or surgical delay. The gains observed are consistent with the impact of targeted education, enhanced adherence and reinforced self-management rather than pharmacological intensification. Key strengths of this work include the large, real-world cohort and comprehensive evaluation of clinical and socioeconomic factors. However, the retrospective design limits causal inference. Furthermore, regression to the mean may have contributed to the observed HbA1c reductions, particularly given the relatively high median baseline HbA1c. Further studies incorporating a comparator group would help quantify the independent effect of DSN-led intervention. While the physiological link between HbA1c reduction and improved surgical recovery is established in the literature, this study did not directly assess postoperative complications or length of stay, which should be explored in future work.^1^, ^5^ The potential for this pathway to reduce surgical risk should therefore be regarded as evidence-informed inference rather than a demonstrated outcome of the current evaluation.

## Conclusion

A DSN-led preoperative pathway was associated with significant improvements in glycaemic control, particularly for those with higher baseline HbA1c and frequent specialist engagement. These findings support the role of structured, person-centred perioperative diabetes care in complex and socially deprived surgical populations. This model offers a practical, scalable solution to a common perioperative challenge, aligning with the remit of integrated clinical medicine. Future studies should evaluate cost-effectiveness and the optimal integration of diabetes technologies to inform national perioperative care models.

## CRediT authorship contribution statement

**Adhithya Sankar:** Writing – review & editing, Writing – original draft, Methodology, Investigation, Formal analysis, Data curation, Conceptualization. **Mariyah Ahmed:** Writing – review & editing, Writing – original draft, Methodology, Investigation, Data curation. **Mohammed S.O. Ahmed:** Writing – review & editing, Writing – original draft, Investigation, Formal analysis, Data curation. **Hood Thabit:** Writing – review & editing, Supervision, Methodology, Investigation, Formal analysis, Data curation, Conceptualization. **Jacqueline Toombs:** Writing – review & editing, Methodology, Investigation, Conceptualization.

## Ethics approval and consent to participate

As an audit and service evaluation, no ethical approval was required.

## Funding

This research did not receive any specific grant from funding agencies in the public, commercial, or not-for-profit sectors.

## Declaration of Competing Interest

HT receives consulting fees and speaker honoraria from Eli Lilly, reports having received research support from Dexcom Inc and participated in advisory groups for Medtronic and Roche Diabetes. MSA, MA, and AS have no conflicts of interest to disclose.

## Data Availability

The data that support the findings of this study are available from the corresponding author, Dr Adhithya Sankar, upon reasonable request.

## Footnotes

Appendix A Supplementary data associated with this article can be found in the online version at doi:10.1016/j.clinme.2026.100619.
