# Supplementary material for: Structured, person-centred glycaemic optimisation before surgery: The Manchester IP3D experience
Source: Clin Med (Lond). 2026 Jul 23;26(5):100619. doi: 10.1016/j.clinme.2026.100619 (PMC13427390; doi:10.1016/j.clinme.2026.100619)
Supplement: Supplementary file 1 — Supplementary material [file mmc1.docx]

| **Table A1: Diabetes treatment change following preoperative nurse review** | | |
| --- | --- | --- |
| **Diabetes Treatment** | **Pre nurse review (%)** | **Post nurse review (%)** |
| Diet only | 5.1 | 1.8 |
| OHA only | 35.0 | 29.9 |
| OHA + GLP-1 | 5.1 | 5.9 |
| GLP1 | 0.3 | 0.3 |
| GLP1 + insulin | 0.3 | 0.3 |
| Insulin only | 27.9 | 28.6 |
| Insulin + OHA | 22.3 | 27.6 |
| Insulin + OHA + GLP-1 | 4.1 | 5.7 |
| OHA = Oral Hypoglycaemic Agent; GLP-1 = Glucagon-Like Peptide-1 analogue | | |

| **Table A2: Predictors for change in perioperative HbA1c following perioperative nurse intervention** | | | | | |
| --- | --- | --- | --- | --- | --- |
| **Predictor** | **B (Unstd.)** | **SE** | **β (Std.)** | **t** | **p-value** |
| Baseline HbA1c^*^ | –0.384 | 0.056 | –0.423 | –6.889 | <0.001 |
| Number of nurse visits^*^ | –0.803 | 0.228 | –0.255 | –3.519 | <0.001 |
| HbA1c repeat interval | –0.012 | 0.073 | –0.013 | –0.167 | 0.867 |
| Sex | –0.040 | 2.344 | –0.001 | –0.017 | 0.986 |
| Age | –0.065 | 0.083 | –0.046 | –0.787 | 0.432 |
| BMI | 0.308 | 0.297 | 0.122 | 1.038 | 0.301 |
| Standardised IMD Rank | –18.991 | 14.184 | –0.759 | –1.339 | 0.182 |
| Standardised IMD Decile | 23.733 | 17.047 | 0.787 | 1.392 | 0.165 |
| Diabetes Type | –2.832 | 2.573 | –0.074 | –1.101 | 0.272 |
| Change in diabetes treatment | –0.063 | 2.839 | –0.001 | –0.022 | 0.982 |
| Sensor user | 0.520 | 3.303 | 0.013 | 0.157 | 0.875 |
| Pump user | –6.288 | 5.126 | –0.084 | –1.227 | 0.221 |
| HCL user | 19.738 | 14.519 | 0.078 | 1.359 | 0.176 |
| New Sensor Start | –1.550 | 4.967 | –0.021 | –0.312 | 0.755 |
| HbA1c to surgery time (weeks) | –0.059 | 0.055 | –0.069 | –1.076 | 0.283 |
| Model performance | Adjusted R^2^ = 0.339 | |  |  | <0.001 |
| *statistically significant  B (Unstd.) = The unstandardised regression coefficient  SE = Standard Error of the unstandardised coefficient  β (Std.) = Standardised coefficient  t = t-statistic (B/SE) | | | | | |
